# Concerns about “BIOFIRE FILMARRAY Meningitis/Encephalitis panel for the aetiological diagnosis of central nervous system infections: a systematic review and diagnostic test accuracy meta-analysis” by *Trujillo-Gomez et al*

**DOI:** 10.1016/j.eclinm.2026.104097

**Published:** 2026-08-18

**Authors:** Kyle Hueth, José Luis Cortes-Cuevas, Lélia Abad, Julien Textoris, Rafael Cantón, Maurizio Sanguinetti

**Affiliations:** a bioMérieux Inc, Salt Lake City, UT, 84104, USA; b bioMérieux SA, Marcy L'Etoile, France; c Servicio de Microbiología, Hospital Universitario Ramón y Cajal and Instituto Ramón y Cajal de Investigación Sanitaria (IRYCIS), Madrid, Spain; d CIBER de Enfermedades Infecciosas (CIBERINFEC), Instituto de Salud Carlos III, Madrid, Spain; e Dipartimento di Scienze di Laboratorio ed Ematologiche, Fondazione Policlinico Universitario Agostino Gemelli IRCCS, Roma, Italy

With great interest, we read the meta-analysis by Trujillo-Gomez et al. on the diagnostic performance of the BIOFIRE® FILMARRAY® Meningitis/Encephalitis (ME) Panel.^1^ We acknowledge the importance of this work and its relevance to clinical practice. However, we were surprised to observe a significant discrepancy in sensitivity compared to the manufacturer's claims and previously reported performance, including those referenced in the meta-analysis.^2^, ^3^, ^4^ In this context, we aimed to reproduce the meta-analysis using the authors' dataset. Unfortunately, the supplementary material only included true positive (TP) and false positive (FP) values. As sensitivity estimates could not be recalculated, two authors from our group independently reviewed all the studies included in the meta-analysis to recalculate sensitivity and specificity values for reference 1. Our evaluation revealed discrepancies in sensitivity estimates for several bacteria species (shown in Table 1), which may stem from methodological choices in the meta-analysis.

**Table 1:** Comparison of sensitivities and specificities for reference test 1 with re-extracted data and fixed- and random-effect model.

CI, confidence interval.

Indeed, we identified that the authors have used a 0.5 continuity correction for zero-event cells with the R software (version 4.5) “mada” package. With this parameter, we were able to replicate the results of the authors. While the impact of using the continuity correction is a well-known statistical issue, some authors may underestimate its influence on point estimates.^5^^,^^6^ It is important to consider that most of the studies included in the meta-analysis by Trujillo et al. have very small numbers of individual bacterial or viral results, and a majority of them have at least one zero cell in the 2 × 2 diagnostic performance table (63 of the 73 article results for individual targets, i.e. 86% have zero cells). Meta-analysis results with continuity correction are most impacted when the sample size at the study level is small.^6^ While the sample size for specificity estimation (reference negatives) is at least 20 for each individual target, the sample size for sensitivity estimation (reference positives) is much smaller; in the reviewed articles, 58 of the 73 article results for individual targets have less than 5 reference positive results, and 32 have 1 or 0 reference positive results. Therefore, sensitivity estimates at the individual target level may be substantially impacted by the use of continuity correction methods. A more appropriate method of meta-analysis for proportions with sparse data is generalized linear mixed models (GLMMs). These models allow for the unbiased estimation of both within study and between study variation.^7^ Using the extracted data, we applied GLMMs via the R (version 4.5) “meta” package to determine fixed-effect and random-effect estimates of sensitivity and specificity.^8^ Notably, we observed a substantial increase in sensitivity estimates across all bacterial species, ranging from 8.2 to 33.5 percentage points (Table 1). Forest plots corresponding to these estimates are provided in the Supplementary Figure S1 and can be compared to the forest plots provided in the original paper Supplemental Material Appendix 5.

In addition, although we included the same studies for each species as Trujillo et al., we noted that several eligible studies were not included in their analysis for some species. These include Lindstrom et al., and Tarai et al. for *Haemophilus influenzae*, *Listeria monocytogenes*, *Escherichia coli*, *Neisseria meningitidis*; Lindstrom et al., Tarai et al. and Piccirilli et al. for *Streptococcus agalactiae*; Lindstrom et al., Tarai et al., Piccirilli et al. and Lopez-amor et al. for *Streptococcus pneumoniae*.^9^, ^10^, ^11^, ^12^ This selective inclusion may also influence the overall estimates of diagnostic accuracy.

In summary, our reassessment suggests that the reported findings may not fully reflect the diagnostic performance of the BIOFIRE® FILMARRAY® ME Panel. This could influence clinical interpretation and decision-making, particularly in central nervous system infections, where the etiologic diagnosis is challenging and alternative diagnostic tools are limited. We hope these observations will contribute to a constructive scientific dialogue and encourage the authors to recalculate sensitivity and specificity using an alternative analytical approach that allows for a more objective assessment of diagnostic performance.

## Contributors

Kyle HUETH, José Luis CORTES-CUEVAS: data extraction, analysis & interpretation of the findings.

Lélia ABAD: interpretation of the findings & first and final draft writing.

Julien TEXTORIS, Rafael CANTÓN, Maurizio SANGUINETTI: supervision and critical revision.

## Declaration of interests

Kyle HUETH, José Luis CORTES-CUEVAS, Lélia ABAD, Julien TEXTORIS are employees at bioMérieux. Julien TEXTORIS owns shares in bioMérieux.

Rafael CANTÓN has participated in educational programs exposored by BD, BioMerieux and had research programs founded by BioMerieux, Cepheid, FastInove, Quantamatrix and Resistell.

Maurizio SANGUINETTI has received honoraria for presentations to Congresses by Alifax and Biomerieux.

## Figures and Tables

**Supplementary Data 1:**
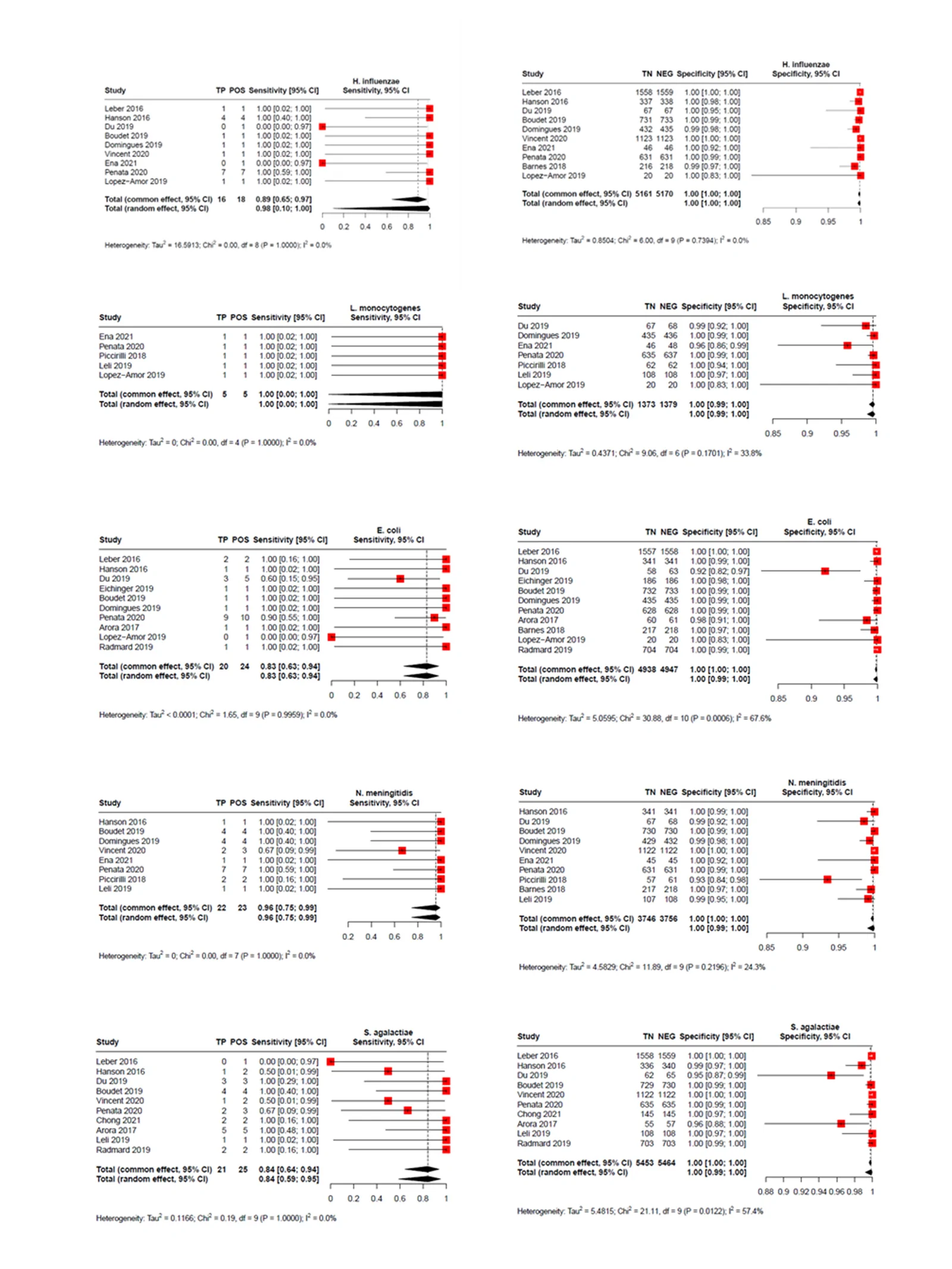


**Supplementary Data 2:**
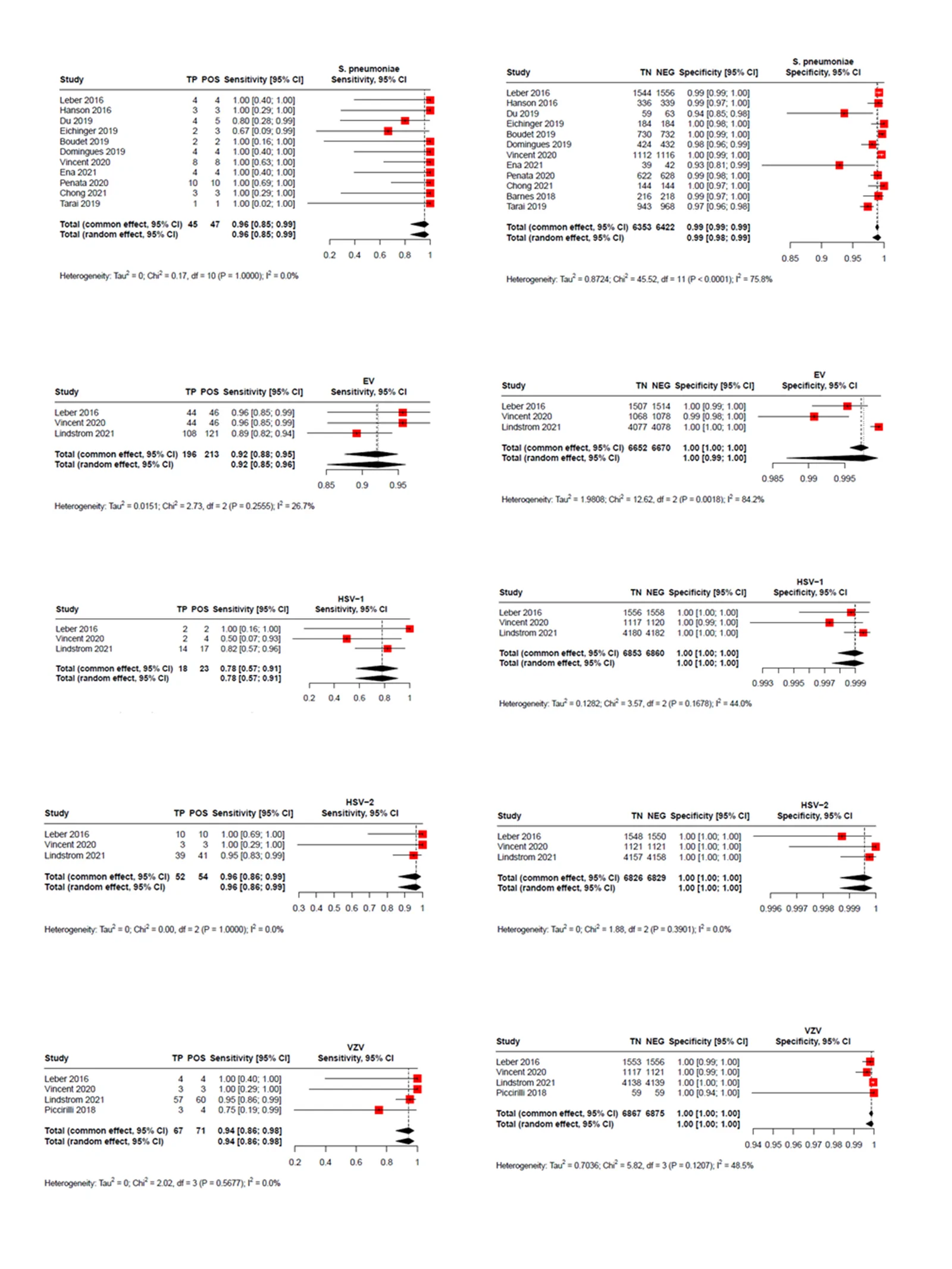


## Appendix A Supplementary data

Supplementary Caption
