# Supplementary material for: Concerns about “BIOFIRE FILMARRAY Meningitis/Encephalitis panel for the aetiological diagnosis of central nervous system infections: a systematic review and diagnostic test accuracy meta-analysis” by Trujillo-Gomez et al
Source: eClinicalMedicine. 2026 Aug 18;99:104097. doi: 10.1016/j.eclinm.2026.104097 (PMC13521013; doi:10.1016/j.eclinm.2026.104097)
Supplement: Supplementary file 1 [file mmc1.docx]

**Supplementary Figure S1.** Forest plots of sensitivity and specificity estimates for individual pathogens. Sensitivity plots display true positive counts (TP) and total positives (POS), and specificity plots show true negative counts (TN) and total negatives (NEG) associated with 95% confidence interval (95%CI). Estimates are provided using both common effect and random effects models. Heterogeneity was assessed with Tau², Chi², I².
